# End Organ Affection in Sickle Cell Disease

**DOI:** 10.3390/cells13110934

**Published:** 2024-05-29

**Authors:** Tanvi Bathla, Saran Lotfollahzadeh, Matthew Quisel, Mansi Mehta, Marina Malikova, Vipul C. Chitalia

**Affiliations:** 1 Renal Section, Department of Medicine, Boston University School of Medicine, Boston, MA 02118, USA; tbathla@bu.edu (T.B.); slotfoll@bu.edu (S.L.); mquisel@bu.edu (M.Q.); 2 Saint Vincent’s Medical Hospital, Worcester, MA 01608, USA; mansi.mehta@stvincenthospital.com; 3 Department of Surgery, Boston University School of Medicine, Boston, MA 02118, USA; marina.malikova@bmc.org; 4 Veterans Affairs Boston Healthcare System, Boston, MA 02118, USA; 5 Institute of Medical Engineering and Science, Massachusetts Institute of Technology, Cambridge, MA 02139, USA; 6 Center of Cross-Organ Vascular Pathology, Department of Medicine, Boston University Medical Center, Evans Biomedical Research Center, X-530, Boston, MA 02118, USA

**Keywords:** sickle cell disease, end-organ damage, acute chest syndrome, thrombosis, sickle cell nephropathy

## Abstract

Sickle cell disease is an orphan disease affecting ethnic minorities and characterized by profound systemic manifestations. Although around 100,000 individuals with SCD are living in the US, the exact number of individuals is unknown, and it is considered an orphan disease. This single-gene disorder leads to red blood cell sickling and the deoxygenation of hemoglobin, resulting in hemolysis. SCD is associated with acute complications such as vaso-occlusive crisis, infections, and chronic target organ complications such as pulmonary disease and renal failure. While genetic therapy holds promise to alter the fundamental disease process, the major challenge in the field remains the target end organ damage and ways to mitigate or reverse it. Here, we provide an overview of the clinical manifestations and pathogenesis with a focus on end-organ damage and current therapeutic options, including recent FDA-approved stem cell and gene editing therapies.

## 1. Introduction

Sickle cell disease (SCD), an autosomal recessive disorder, is the most common inherited blood disorder worldwide. In SCD, the fundamental defect is a point mutation in the globin chain where the 17th nucleotide is changed from thymine to adenine, and correspondingly, the sixth amino acid, glutamic acid (hydrophilic), is replaced by valine (hydrophobic). The homozygous expression of the mutation results in the full-blown SCD, while the expression of a single copy of the gene results in the sickle cell trait. Sickle hemoglobin is a structural variant of normal adult hemoglobin (HbA:α_2_β_2_). The hemoglobin molecule has 2α and 2β subunits, each consisting of a heme moiety to transport an oxygen molecule. The polymerization of the two mutant sickle-globin subunits results in the crescent or sickled shape of erythrocytes; hence, it is called SCD. The sickled Hb (HbS) further affects RBC viability and adhesiveness and leads to secondary consequences like hemolysis, endothelial damage, pain crises, and the thromboses of blood vessels affecting major organs like the lungs, brain, kidneys, and bones [1]. HbSC refers to the sickle cell trait and shows milder manifestations. This review focuses on SCD and not a combination of SCD with other hemoglobinopathies.

## 2. Epidemiology 

SCD is an understudied lifelong disease impacting underserved minorities in the United States and the entire world. Approximately 230,000 neonates are born with SCD annually in Africa, 43,000 in Southeast Asia, 13,000 in America, 10,000 in the Eastern Mediterranean region, 3500 in Europe, and 4 in the West-Pacific [2]. A systematic analysis from the Global Burden of Disease study in 2021 revealed that the total births of SCD babies increased globally by 13.7% [3].

### 2.1. Prevalence

In terms of prevalence, the highest cases of HbS in the world are present in Sub-Saharan Africa, followed by the Middle East and the Indian Subcontinents [4]. In the United States, around 100,000 individuals are currently living with sickle cell disease, the majority of whom are African American [5]. As per the CDC data, in the USA, SCD occurs in 1 out of every 365 Black or African American births and 1 out of every 16,300 Hispanic-American births, and around 1 in 13 African-American babies are born with the sickle cell trait [6]. In a study conducted by Campbell et al. [7], they analyzed the racial and ethnic backgrounds of SCD patients. They found that in the United States, patients were mostly African American (71%), followed by Caribbean (13%) and West African (10%). In contrast, in Europe, West Africans comprised 73% of the SCD patients followed by Europeans (10%), people from the Caribbean (8%), and Central Africans (8%). Pokhrel et al. [8] analyzed the NIS database for 74,814 patients hospitalized for sickle cell disease. Blacks constituted the majority of the patient population (93.4%) followed by Hispanics (4.8%) and Whites (1.8%) (Table 1, Table 2 and Table 3).

According to a systematic review from Global Burden of Diseases-2021, the prevalence of SCD increased globally by 41.4% [3]. In the US, there is a dearth of studies on SCD in adults, and till now, there are no published national population estimates of the prevalence of SCD including the growing Hispanic population. As per the Health Care Policy and Research (AHCPR), the SCD population prevalence estimate was ~115,442 according to the 2008 census data [5].

Due to the racial and ethnic composition in different states in the US, there is a difference in prevalence rates. In a study by Kathryn L. Hassel [5], the total birth cohort-SCD prevalence estimation from 2005 to 2007 from 37 US states was found. It significantly varied by state; SCD occurred in 1:400–1:600 births in states with at-risk populations in Mississippi and the District of Columbia but the figure was 1:20,0000–1:30,000 of all births in the low-risk states such as Utah and South Dakota. 

### 2.2. Gender Variation

Although SCD is transmitted as an autosomal recessive disorder, certain studies have found gender-related differences in SCD mortality and morbidity in adult patients. A study conducted by Platt et al. [9] found that females had a longer lifespan than males, with a mean death age of 42 years for males and 48 for females. This finding was further supported by a study of pediatric patients by Ceglie et al. [10] who retrospectively studied the clinical records of 39 pediatric patients with SCD over ten years and found gender-related differences. Interestingly, the mean vaso-occlusive pain crisis episodes were 1.6 in the male population as compared to 0.6 in the female population. Moreover, cardiac anomalies were present in 10 males, 3 females, and 4 male children had osteomyelitis as compared to 1 female child. They concluded that gender plays a role in the pathogenesis of the disease, with a more aggressive course in the males. Another study by Payne et al. [11] found that SCD-related deaths among male individuals were 9% greater than in females, although the exact reason for this gender variation needs to be determined.

### 2.3. Mortality Rates

SCD is associated with end-organ damage and significant complications, which further lead to a reduction in life expectancy [12].

In younger patients, the main cause of mortality is septicemia, in contrast to the older population, wherein it is multi-organ damage. Chronic lung disease with pulmonary hypertension and cor pulmonale, followed by chronic renal failure, is the leading cause of death in older patients [13]. Payne et al. [11] studied the trends in sickle cell disease-related mortality in the United States from 1979 to 2017 and found 25,665 SCD-related deaths among black people. However, the median age at death dramatically increased from 28 years to 43 years, and the average annual death rate among children under the age of 5 years declined from 2.05 per 100,000 in 1979 to 0.47 per 100,000 from 2015 to 2017. They also found a similar trend with acute events such as infection and cerebrovascular events as the cause of death in the younger age group and chronic cardiac and renal complications among the older age group. However, the most significant shift was noted in the reduction in SCD-related deaths by acute infections by 21% in children less than five years old [11]. Kathryn L. Hassel [5] found that there was a shift toward death at older ages, with mortality rates reduced by 39%. 

This improvement can be ascribed to the advances in treatment, mainly the usage of prophylactic antibiotics (to prevent infection), awareness about immunizations, screening, and follow-ups in newborns. 

Studies have shown that the mortality rates and end-organ damage correlate with the percentage value of HbF. Platt et al. [9] described that the risk of early death inversely correlated with the level of fetal hemoglobin (HbF). They also found an enhanced survival of patients with HbF above the 75th percentile. These findings were further strengthened by Powars et al. [14], who found reduced incidence rates of sickle cell crisis, chest syndrome, and hospitalizations for patients with HbF > 20%. Fitzhugh et al. [15] noted a statistically significant association between the use of hydroxyurea (an agent known to increase HbF levels, vide infra) and decreased mortality.

Despite all the latest interventions, the life expectancy has reached around 55 years, which is still significantly lower than that of the normal population of the same ethnicity [16].

## 3. End-Organ Damage

**Figure 1:**
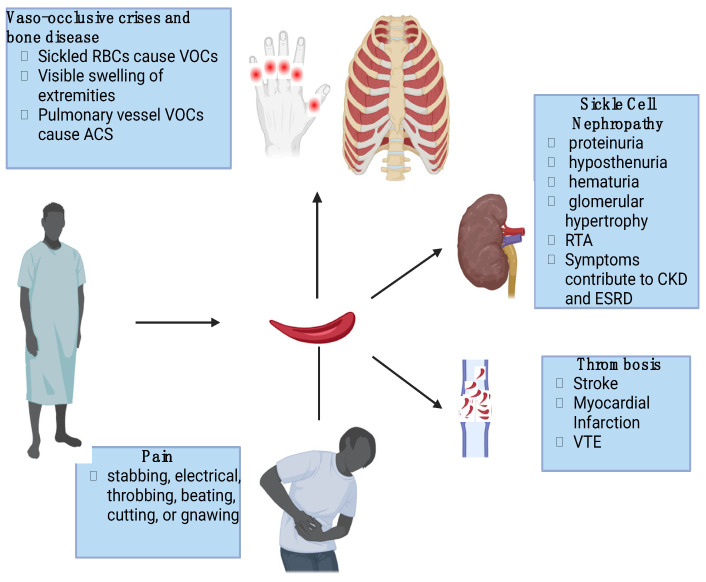
Clinical manifestations of SCD.

SCD is a complex genetic disorder involving multiple organs and systems (Figure 1).

### 3.1. Vaso-Occlusive Crises and Bone Disease

Acute episodes of pain, referred to as vaso-occlusive crisis, are the most common morbidity associated with SCD and the leading cause of hospitalization in around 95% of patients [17,18]. Recurrent episodes have a detrimental effect on the quality of life as compared to organ damage [19].

Pain commonly occurs in the lower back, joints, and extremities. Recurrent vaso-occlusion and infarction of the articular surfaces leading to avascular necrosis are seen in 12–15% of children with SCD [20]. These recurrent episodes drastically affect the quality of life of these patients [21]. Patients describe pain as sudden in onset and sharp and throbbing. There is a prodromal phase of 1–2 days, with a maximum pain intensity on day 3, which lasts for 6–7 days and then wears off. Most patients require hospital admissions, and the length of stay varies with the age group. The average length of stay for children is 4.4 days [22]; for adults, it is 2.3 days in the emergency room and 11.7 days in the inpatient setting [23]. 

Further, the co-existence of VOCs with acute chest syndrome (ACS) is associated with a high mortality rate in patients with SCD [9].

### 3.2. Acute Chest Syndrome

Acute chest syndrome (ACS) is one of the most dreadful complications of SCD and is characterized by a new radiodensity on the chest radiograph and is accompanied by one of the following: (1) fever; (2) hypoxemia; (3) tachypnea; and (4) cough, chest pain, rales, and wheezes [24]. It mostly coincides with vaso-occlusive events. Patients with SCD and asthma are at a two to four times higher risk of developing acute chest syndrome [25].

ACS is the most common cause of ICU admissions and premature death and the second most common cause of hospitalization in SCD patients [26]. Moreover, around 29% of all SCD patients suffer from at least one episode of ACS, out of which half will experience more than one throughout their lifetime [27]. Though it can affect any subtype of SCD, it is most commonly found in people with HbSS (12.8 per 100 patient-years), followed by HbSC (5.2 per 100 patient-years) [27].

### 3.3. Sickle Cell Nephropathy

The effect of SCD on the kidney is grouped under sickle cell nephropathy (SCN). SCN is a broad entity presenting with hematuria, proteinuria, hyposthenuria, renal papillary necrosis, renal tubular disorders, acute and chronic kidney injury, sickle cell glomerulopathy, and renal medullary carcinoma. Approximately 5% of individuals with SCD (SCD) suffer from chronic kidney disease (CKD) [28]. The prevalence of microalbuminuria is 26.5% starting at the age of ~7 years and rises in adults with a prevalence of about 46% in the second decade of life [29]. Interestingly, the prevalence of microalbuminuria is more common in SS disease as compared to other sickling disorders, and it was found in 44% of adults with HbSS in comparison to 23% with HbSC [30].

#### 3.3.1. Hyposthenuria

The loss of concentration ability results in dilute urine. It arises due to defective medullary tonicity and is evident as early as three years of age, and its risk increases with age [31].

#### 3.3.2. Hematuria

Asymptomatic hematuria is one of the most common findings in SCD [32]. Gross hematuria episodes are mostly self-limited. Interestingly, the left kidney is more commonly involved due to increased venous pressure caused by the greater length of the left renal vein. Hematuria is mainly caused by renal papillary necrosis. As discussed above, dehydration, acidosis, decreased oxygen tension, and high osmolarity precipitate red blood sickling in the renal medulla creating an ideal condition for papillary necrosis. Accompanying symptoms include flank pain, nausea, and vomiting. In a few cases, hematuria is caused by renal medullary carcinoma and leads to mortality within two years of diagnosis [33].

#### 3.3.3. Proteinuria

Proteinuria is prevalent in 30% of patients, with its incidence increasing with age [34]. Nephrotic syndrome (greater than 3.0 gms of urine protein/day/1.73 sqm body surface area) is found in 40% of patients with SCN [34] and is a predictor of progression to chronic renal failure [35,36].

#### 3.3.4. Chronic Kidney Disease (CKD) and End-Stage Renal Disease (ESRD) 

The listed renal manifestations of SCN, such as glomerulopathy, hematuria, and tubular dysfunction, all contribute to ESRD. However, proteinuria and CKD are seen in approximately 11.6% of patients with SCD, and the median age of onset is 37 years [13]. Proteinuria associated with glomerulosclerosis leads to CKD and ESRD.

The presence of renal failure varies from 5 to 18% in SCD patients [33]. Platt et al. [8] reported an 18% mortality rate in adult SCD patients, with 40% of these (7.6% of the total) presenting with overt renal failure. Another study conducted by Blake et al. [37] reported a mortality rate of 38% due to CKD in SCD patients. Similarly, Hamideh et al. [38] attributed renal dysfunction as a cause of death in 16.4% of patients with SCD.

#### 3.3.5. Glomerular Hypertrophy 

Increased renal growth initiates in infancy when it correlates with hyperfiltration [39].

#### 3.3.6. Renal Tubular Acidosis

Notably, impaired medullary perfusion can lead to distal nephron dysfunction, which manifests as an acidification defect and impaired ability to secrete potassium. Certain studies have demonstrated the presence of distal renal tubular acidosis (RTA). In SCD, around 40% of patients present with normokalemic RTA. Hyperkalemic hyperchloremic metabolic acidosis is seen in patients with SCD due to a type IV RTA defect or selective aldosterone deficiency [40]. Thus, it can be inferred that the co-existence of SCD and renal failure results in severe detrimental outcomes as compared to when the disease occurs alone.

## 4. Thrombosis

### 4.1. Stroke

The incidence of stroke is highest in children, being 1.02% per year between the ages of 2 and 5 years old [41]. Notably, about two-thirds of children are likely to experience another episode of stroke within the first 2 to 3 years of their previous episode [42].

It has been reported that the incidence of first stroke in the African-American population younger than 35 years old is 500 to 1280 per 100,000 person-years in SCD, which is strikingly high as compared to the non-SCD group, where it is 12 per 100,000 person-years [43]. Ischemic strokes affect both younger and older patients, whereas hemorrhagic strokes have the highest prevalence between 20 and 29 years of age with SCD [41].

Overt strokes mainly present as weakness, dysarthria, seizures, sensory deficits, and headaches, whereas silent cerebral infarcts are associated with cognitive deficits and impaired academic performance [44]. Most of these children have persistent cognitive deficits ranging from language problems and reduced IQ to moderate mental retardation [45].

### 4.2. Cardiovascular Complications

Myocardial infarction and coronary microvascular disease are prevalent in SCD, though there are very few studies describing their incidence. In a study conducted by Kaur et al. [46], they found that the troponin level was elevated in 18% of SCD patients, indicating that SCD patients experience myocardial injury when presenting with chest pain.

Among all cardiovascular complications, pulmonary hypertension is a frequent and serious complication in adults with SCD. Its prevalence (according to echocardiography) is around 10 to 30% among patients with SCD [47] and falls into category 5 of pulmonary hypertension under “Pulmonary hypertension with unclear or multiple etiologies” [48]. Castro et al. [49] followed up SCD patients for 9 years post-right heart catheterization and reported a 55% mortality rate in patients with pulmonary hypertension as compared to 21.4% without pulmonary hypertension.

### 4.3. Venous Thromboembolism

Due to the association between hypercoagulability and SCD, venous thromboembolism is a known complication of SCD. The median age at first presentation of VTE is 30 years old, and it is prevalent in 25% of SCD patients [50]. The Cooperative Study of SCD revealed an 11.3% cumulative incidence of VTE by the age of 40 [50]. It is noteworthy that the incidence rates of VTE in SCD patients are similar to those with a family history of thrombophilia [50].

## 5. Pain

Severe intermittent acute pain is the most common complication of SCD [51] and is described by patients as deep, stabbing, electrical, throbbing, beating, cutting, gnawing, or generalized toothache. Older patients suffer from chronic back pain due to vertebral collapse because of osteopenia and osteoporosis [21].

## 6. Molecular Pathogenesis of Lung Damage in SCD

In the following section, we summarize the mechanisms involved in pulmonary hypertension in SCD. Several studies have demonstrated the different pathways responsible for pulmonary hypertension in SCD patients.

### 6.1. P-Selectin

Ghosh et al. [43] studied the role of P-selectin in heme-induced acute lung injury using a SCD (SS mouse) mouse model. They infused either a functional blocking monoclonal murine anti-P selectin antibody or control IgG into SS mice, which was then followed by the induction of acute lung injury using purified heme. They observed that five of six SS mice pre-treated with the anti-P selectin antibody did have evidence of lung injury, whereas all the control SS mice had extensive lung damage on post-mortem and died due to severe ischemia. 

They also found that heme infusion led to mortality in wild-type mice irrespective of the presence of P-selectin on the vascular endothelium, further strengthening the fact that continuous intravascular hemolysis leads to inflammation in ACS. Heme was shown to induce severe hypoxemia, lung damage, and pulmonary edema in the presence of endothelial P-selectin in wild-type mice. Furthermore, they found that P-selectin potentiated extracellular heme-induced inflammation by releasing more heme into the circulation by a mechanism involving enhanced adhesions of sickle erythrocytes [52].

### 6.2. Platelet

Intravital microscopy of the lung in transgenic humanized SCD mice has been used by Bennewitz et al. [53] to track acute vaso-occlusive events that occur after a dose of systemic lipopolysaccharide to trigger SCD-related events. They noticed neutrophil–platelet aggregates causing cellular microembolism of precapillary pulmonary arterioles. A function-blocking P-selectin antibody reduced the neutrophil–platelet interactions in SCD blood and resolved pulmonary arteriole microembolism in SCD mice. These findings proved that the generation of neutrophil–platelet aggregates in lung arterioles has a role in SCD-related lung vaso-occlusion [53]. 

Anea et al. [54] conducted an autopsy investigation of 20 SCD cases, 10 of which died from ACS and the rest from causes unrelated to ACS. They detected a platelet-mediated occlusion in 3 out of 10 SCD patients who succumbed to ACS. Interestingly, these occlusions were associated with younger age and higher platelet counts during the acute stage, which significantly declined as ACS worsened. Moreover, the von Willebrand factor (vWF) was significantly deposited in the endothelium where thrombi were found, and massive vWF aggregates were also found attached to the endothelium. The authors suggested that a significant sequestration of platelets in the pulmonary vasculature could be a possible mechanism of ACS in these patients. These findings support applications for anti-platelet therapy in ACS [54].

Another study by Jimenez et al. [55] used quantitative microfluidic fluorescence microscopy (qMFM) to study the relationship between the glycoprotein Ibα inhibitor (CCP-224), a PEGylated form of the OS-1 peptide and neutrophil–platelet aggregation in SCD human blood flowing through microfluidic channels. They demonstrated that, when used in vascular mimic flow circumstances, the GPIb antagonist, CCP-224, is a strong inhibitor of neutrophil–platelet aggregation in the blood of SCD patients [55].

### 6.3. Signal Pathways

IL-18 is a pro-inflammatory and fibrotic cytokine. Alpha-L-Fucosidase 2 (FUCA2) is involved in the immune response, signal transduction, early embryogenesis, apoptosis, and the adhesion of leucocytes. Duarte et al. [56] discovered the association of IL-18 and FUCA2 with diastolic dysfunction in African Americans with SCD using genome-wide analysis. Transcriptome data from both patient populations indicated seven genes related to diastolic dysfunction, and three of these genes were verified by mice SCD myocardial expression. Expression quantitative trait loci(eQTLs) and genetic correlations were found in FUCA2 and IL-18 [56]. 

These observations were further strengthened by Novelli et al. [57], who generated chimeric animals using bone marrow transplant experiments from the Berkeley sickle cell model and found an increase in the circulating plasma levels of TSP1 (thrombospondin) via binding to CD47 receptors and stimulating reactive oxygen species (ROS) production in sickle mice. Thrombospondin-1 is a plasma protein that promotes vascular pathology by inhibiting the vasodilatory, anti-adhesive, and hemostatic effects of the nitric oxide and vascular endothelial growth factor (VEGF) signaling pathways in the vasculature via CD47 receptors [58]. They found that TSP1 and CD47 expressions increased with age in SCD lungs compared to controls. Moreover, the IF of lung sections from patients with SCD-associated pulmonary hypertension also revealed increased levels of CD47. Furthermore, they performed an open-chest hemodynamic assessment of 2–8-month-old male sickle mice and found that they developed pulmonary hypertension and right heart failure and had a higher end-diastolic volume. Interestingly, in the lungs of Berkeley SCD mice and SCD-associated pulmonary hypertension patients, TSP1 and CD47 were elevated. Treatment with TSP1 enhanced the production of ROS in human pulmonary artery endothelial cells, which was suppressed by CD47 inhibition. Supporting the role of CD47, the authors further demonstrated that hereditary CD47 deficiency reduced SCD-associated pulmonary hypertension due to a reduction in ROS levels [57]. 

Menon et al. [59] discovered that the levels of lipid peroxidation and ferroptosis indicators were upregulated in SCD animals with ongoing heme oxygenase 1 (Hmox1) overexpression and iron overload. These abnormalities were reversed by hemopexin treatment. Additionally, ferroptosis inhibitors reduced cardiomyopathy, whereas erastin, a ferroptosis inducer, increased the heart damage in SCD patients and caused cardiac ferroptosis in mice that were not sick. Finally, in SCD animals, Hmox1 inhibition lowered cardiac ferroptosis. They concluded that excess heme upregulates heme oxygenase-one, increasing the cardiac ferroptosis in SCD mice models and that ferroptosis is a fundamental cause of cardiomyopathy in SCD [59].

Another study conducted by Reichel et al. [60] found the role of Ccl2 and Ccl3 with neutrophil recruitment and postulated that platelet-activating factor (PAF) and leukotriene B-4 (LTB4) overexpression was a mechanism by which CCL2 and CCL3 recruited leukocytes, particularly neutrophils in SCD mice. The neutrophils firmly attach to the endothelium and become activated. Leukocytes that are in a state of arrest have their CD11b upregulated, which allows them to interact with platelet-expressed GPIb enhancing thrombogenesis [60]. 

## 7. Mechanism of SCD Nephropathy 

As discussed previously, due to the high rate of oxygen consumption, the kidney is particularly affected by SCD. Renal failure is the leading cause of death in SCD patients with clinically diagnosed chronic organ failure [9]. The pathologies of sickle cell nephropathy are manifested in both the medullary and cortical regions, leading to impaired renal physiology.

### 7.1. Medullary Region Pathogenesis

The susceptibility of the medullary region to SCD may be partly attributed to the hyperosmolar, acidotic, and hypoxic milieu of the renal medulla, as well as the slow blood flow in the vasa recta [61]. Hyperosmolarity in the renal medulla dehydrates RBCs, increasing the intracellular HbS concentration, and hypoxia and acidosis further reduce hemoglobin’s affinity to oxygen, promoting HbS polymerization. A slow blood flow in the vasa recta exacerbates the HbS polymerization by increasing the transit time of RBCs, resulting in higher frequencies of vascular occlusion [61]. Ischemia caused by vascular occlusion and subsequent ischemia–reperfusion damage, combined with systemic inflammation, contribute to SCN [61]. Besides the vascular damage in the medullary region, cortical hyperperfusion is noted in SCD, which manifests as glomerular hypertrophy and proteinuria [62]. 

### 7.2. Endothelial Dysfunction

At the molecular level, endothelin-1 (ET-1) and sVEGFR1 are associated with SCN; both can augment endothelial dysfunction [63,64]. In SCD patients, ET-1 secretion by endothelial cells is elevated because of hypoxia, shear stress, angiotensin II, thrombin activation, and inflammatory cytokines. ET-1, upon binding to its receptor endothelin receptor A (ETA), results in vasoconstriction, inflammation, mitogenesis, and nociception [6]. Treatments using ETA antagonists in transgenic sickle cell mice partially restore renal function, alleviating glomerular oxidative stress, proteinuria, and the urinary excretion of kidney injury molecule 1 (KIM-1), a marker of tubular injury [64,65]. In addition, ET-1 is shown to promote renal fibrosis, and increased ET-1 also causes dehydration through its antagonizing effect against vasopressin [66]. 

sVEGFR1 is shown to be elevated in SCD patients, and leads to decreased nitric oxide (NO) production by inhibiting Akt phosphorylation, which is responsible for activating downstream NO synthase [67]. Reduced NO leads to vasoconstriction, promoting vascular occlusion events in SCD patients.

### 7.3. Oxidative Stress

Heme oxygenase 1 (HMOX1) is an enzyme that uses oxygen to catalyze the conversion of heme into biliverdin, carbon monoxide, and iron. It is overly expressed in the kidneys of transgenic sickle cell mice compared to control mice [68,69]. HMOX1 is considered to be an antioxidant protecting cells from exposure to cell-free hemoglobin and also has anti-inflammatory functions important for vascular health [70]. HMOX1 upregulation in SCD patients is a sign of severe oxidative stress.

Despite high hemolytic stress, haemopexin, a major scavenger of extracellular heme, is downregulated in SCD patients. However, another scavenger, α−1-microglobulin (A1M), is upregulated as a compensatory mechanism in SCD patients. This leads to a substantially higher A1M-to-haemopexin ratio in individuals with SCD compared to healthy controls, which is shown to be a risk factor for acute kidney injury [71].

Angiotensin receptor signaling promotes glomerular pathologic conditions in SCD [72]. Reactive oxygen species (ROS) increase the conversion of oxidized angiotensinogen to angiotensin II (ATII), and ROS levels are elevated in patients with SCD compared with healthy individuals [73]. ATII activates TGF beta signaling, which leads to glomerular sclerosis [74].

Charrin et al. [75] conducted a study on the use of RAGE (receptor for advanced glycation end-products) antagonists to ameliorate the kidney damage in homozygous sickle mice. Under oxidative stress, advanced glycation end-products (AGEs) are generated, and they induce the production of inflammatory cytokines, adhesion molecules, and oxidants. They have been shown to affect renal filtration and induce glomerulopathy [76,77]. The authors experimented on 8–9-week-old Townes mice with human hemoglobin genes and divided them into AA (healthy controls) and SS (homozygous SCD). The RAGE antagonist peptide (RAP) was injected into both groups. They found glomerular hypertrophy and higher interstitial fibrosis in SS as compared to the treated SS group. Furthermore, RAGE inhibition reduced the glomerular area in SS mice, and interstitial fibrosis also improved. While the SS mice of both groups exhibited iron deposits in the kidneys, RAP-SS mice had fewer deposits than the control SS mice. In addition, KIM-1 was lowered by 50% in RAP-SS mice compared to the control mice; however, the levels of KIM-1 in RAP-SS mice were still far greater than the AA mice’s levels.

**Figure 2:**
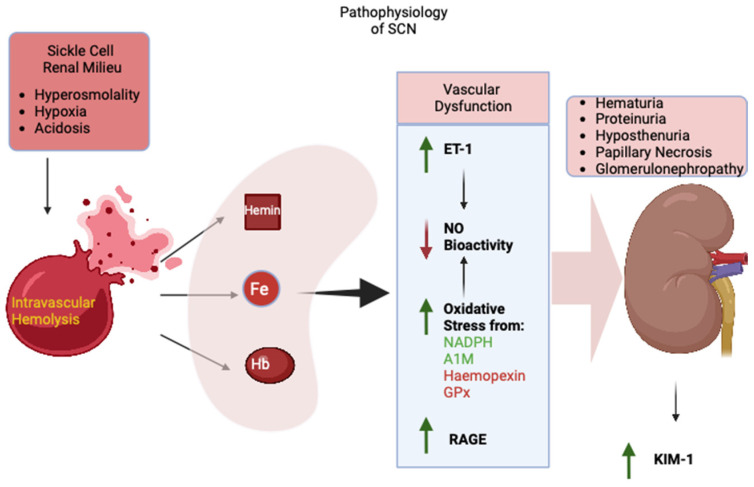
Pathophysiology of SCN. Green arrow: Increased levels; Red arrow: Decreased levels; Black arrow: Leads to.

Interestingly, RAP-SS mice also showed decreased levels of NADPH activity and decreased levels of glutathione peroxidase activity, which ameliorates oxidative stress in RAP-SS mice by the AGE/RAGE signaling pathway. A RAGE blockade was also shown to increase hematocrit, RBC count, and hemoglobin levels and decrease reticulocyte and sickle cell counts in SS mice [75] (Figure 2). 

## 8. Mechanism of Pulmonary Hypertension in SCD

The possible mechanisms for the development of pulmonary hypertension include hemolysis, hypoxia, thromboembolism, and a combination of these. 

### 8.1. Hemolysis 

Intravascular hemolysis leads to the release of cell-free hemoglobin and submicron RBC microparticles containing hemoglobin and heme, and arginase-1 into plasma. These mediators, in turn, inhibit NO signaling and vascular endothelial function [78]. Hemolysis also promotes platelet activation and chronic inflammation, further perpetuating pulmonary vascular occlusion and pulmonary hypertension [79].

### 8.2. Hypoxia

Chronic hypoxia is an important cause of pulmonary hypertension. SCD is characterized by high levels of erythropoietin, which is induced in hypoxia and the activation of hypoxia-inducible factor-α. This phenomenon is responsible for the changes in the mitochondria redox signaling and contributes to the proliferation of pulmonary vascular cells, further occluding the pulmonary vascular and increasing the pressure [80].

### 8.3. Coagulation and Thrombosis

Ataga et al. [81] studied the association of coagulation markers’ activation and inflammation with pulmonary hypertension in SCD. The authors found that the patients with SCD have higher levels of the thrombin–antithrombin complex, prothrombin fragment F1+2, D-dimer, and endothelial soluble vascular endothelial cell adhesion molecule (sVCAM) activation as compared to control subjects. Interestingly, HbSS patients with pulmonary hypertension had higher levels of various inflammatory cytokines (IL-6, 8 and 10) than HbSS without pulmonary hypertension. Furthermore, the markers of hemolysis are positively correlated with coagulation activation and endothelial dysfunction. They inferred that the endothelial dysfunction and coagulation activation were due to hemolysis via the nitric oxide pathway [81]. These studies strongly suggest an intertwined relationship between coagulation, inflammation, and endothelial activation contributing to pulmonary hypertension.

## 9. Mechanism of Thrombosis in SCD

In patients with SCD, the development of large vessel thrombosis is seen mainly due to intravascular coagulation as well as erythrocyte sickling [82,83].

The circulating TF-positive microparticles produced from red blood cells, platelets, endothelial cells, and monocytes may also activate the clotting system with thrombin production in sickle cell patients. Shet et al. [70] found that in sickle cell patients, total blood MPs are elevated during the crisis, and the sickle blood has microparticles derived from endothelial cells and monocytes. Interestingly, some of these microparticles were tissue factor positive [84], supporting them as being a contributory factor in thrombosis.

Westerman et al. [85] showed that in HbSS disease, antiphospholipid antibodies were increased, with IgG phosphatidylserine showing the highest increased levels, whereas protein C (activity) and protein S (activity, total, free antigen) were decreased. Moreover, all measures of coagulation activation were increased. They concluded that the antiphospholipid antibody, IgG-PS, would have contributed to coagulation activation in HbSS disease. This can be attributed to the exposure of the procoagulant, phosphatidylserine, on the red cell shed vesicles and sickle red cell surface, which leads to coagulation activation [85].

These findings are further strengthened by a study conducted by Green et al. [86], who determined protein C activity, fibrinopeptide A, fibrinogen, and β thromboglobulin in SCD patients and found that they had significantly lower concentrations of protein C and higher levels of fibrinogen, fibrinopeptide A, and β thromboglobulin as compared to the control group. However, there was no difference in their levels during the period of crises and the asymptomatic phase in the SCD group [86].

Another study conducted by Nsiri et al. [87] studied the abnormalities of coagulation and fibrinolysis in children and adults with homozygous SCD and a control group. It determined PT, aPTT, and TT ratios (ratios of a patient’s value to standard clotting time) and concluded that PT ratios were significantly increased in SCD patients both during steady state and painful crises. In contrast, aPTT levels were the same in both groups but the values were higher during painful crises. Moreover, fibrinogen levels were the same but TT ratios were surprisingly low in the steady state, and they further declined during the crisis. These results can be explained by the presence of circulating activated clotting factors accelerating thrombin generation. They further found increased levels of D-dimers during the period of crisis. Moreover, increased PAI-1 levels were found in patients with SCD. These findings can be due to the vascular injury caused by the repeated adherence of sickle erythrocytes to the vascular endothelium and their detachment due to the blood flow. Furthermore, infections and inflammation can also lead to increased PAI-1 levels [87].

## 10. Treatment of SCD

After Max Perutz [76] studied the structure of hemoglobin extensively in the 1960s and 1970s, Donald Abraham [77] became a pioneer in the 1970s and 1980s. Using X-ray crystallography and structure-based techniques, their group found several compounds that targeted pockets on the surface of Hb and those binding to the central water cavity of the protein. They employed three different methods of design and produced active compounds that could bind specifically to the surface of Hb near the contact areas of HbS as determined via X-ray crystallography, which resulted in the active meta-disubstituted benzoic acids. Another method was to modify the binding sites of agents so that they could better fit the site, which resulted in phenoxyacetic acids. Lastly, they added a polar side chain to the insoluble anti-gelling agent, p-bromobenzylalcohol, and produced benzoyl acetic acids. They studied their anti-gelling activities and their effect on the allosteric mechanism of Hb. These steps were taken to design several anti-sickling agents in the future.

Despite an understanding of the molecular pathogenesis of the disease, the available treatment options remain limited for SCD.

### 10.1. General

#### 10.1.1. Hydration

Adequate hydration helps in preventing vaso-occlusive crises. Patients are encouraged to drink plenty of fluids during the episodes of VOC and infections, and in some cases, IV fluids are given.

#### 10.1.2. Hydroxyurea

Hydroxyurea is the first FDA-approved drug for SCD and, to date, appears to be the best available therapy for children and adults with SCD due to its safety profile, and ease of oral administration. It increases the concentration of fetal hemoglobin, thereby preventing VOC events, anemia, hemolysis, and chronic organ damage.

### 10.2. Management of End-Organ Damage

#### 10.2.1. VOC and Pain

The mainstay of treatment is the initiation of opioid analgesia (oral, intranasal, subcutaneous, intravenous) at the starting dose of 0.1 mg/kg morphine and repeating the dosage frequency as needed [88]. Acetaminophen and NSAIDs are used as adjuvant therapies. Other adjuvant modalities include hydration, intravenous fluids, acupuncture, yoga, and massage [88]. Additionally, hydroxyurea has been shown to reduce the frequency of painful crises and the need for blood transfusions in adult patients with recurrent moderate to severe painful crises (at least three VOCs during the previous month) [89].

#### 10.2.2. Primary Pulmonary Hypertension

In these individuals, targeted therapies such as phosphodiesterase-5 inhibitors, endothelin receptor antagonists, and prostacyclin analogs may be used to slow disease progression [90].

#### 10.2.3. Renal Complications

In sickle cell glomerulopathy, ACEIs and ARBs remain the mainstay of treatment to reduce proteinuria [91]. Hematuria is mainly treated via adequate hydration and hydroxyurea [92]. In ESRD, chronic hemodialysis and renal transplantation remain the available options [93].

### 10.3. Disease-Modifying Therapies in SCD

Hydroxyurea continues to be the mainstay of treatment for SCD. However, with the recent FDA approval of three disease-modifying drugs, there is some hope in this aspect.

#### 10.3.1. L-Glutamine

L-Glutamine protects against oxidative damage and effectively reduces pain episodes and acute chest syndrome [94].

#### 10.3.2. Crizanlizumab

P-selectin promotes vaso-occlusion by triggering inflammation, adhesion of neutrophils, and platelet activation. Crizanlizumab, a humanized monoclonal antibody, binds to P-selectin and blocks the adhesion molecule’s interaction with its ligand. FDA approval was based on a phase 2 randomized clinical trial, which showed a reduction in pain events when patients were administered with high-dose Crizanlizumab (5 mg/kg dose) [95]. However, the results from the recent global phase 3 study STAND (NCT03814746) [96] indicate no statistically significant difference between crizanlizumab 5 mg/kg or crizanlizumab 7.5 mg/kg and placebo in annualized rates of vaso-occlusive crises (pain crises) or healthcare visits over the first-year post-randomization. Moreover, they reported higher rates for grade > 3 treatment-related adverse events. While further evaluation is ongoing, it has been advised to consider individual benefits and risks while prescribing this drug by physicians [96]. 

#### 10.3.3. Voxeletor 

Voxeletor is an allosteric modifier of HbS that increases the oxygen affinity by reversibly binding to hemoglobin. Its FDA approval was based on a phase 3 randomized clinical trial, which showed an increase in Hb of 1 g/dL after 24 weeks of treatment and reduced anemia severity. Furthermore, it has been shown to reduce indirect bilirubin and reticulocyte percentage [97].

### 10.4. Stem Cell and Gene Editing Therapies

RBC exchange has been used for treating SCD-related complications. Additionally, hematopoietic stem cell transplantation is the only curative therapy for SCD, but a major limitation of its use is the availability of HLA-matched siblings. Gene editing therapy using CRISPR (clustered regularly interspaced short palindromic repeats) has been used in a few clinical trials [98]. Another latest first-in-human study was conducted to silence the BCL11A gene using CRISPR/Cas9 editing and was shown to eliminate VOC in SCD patients [99].

Recently, the FDA approved two milestone treatments, Casgevy and Lyfgenia, representing the first cell-based therapies for the treatment of SCD in patients 12 years old and younger [100]. Casgevy utilizes CRISPR/Cas9 to modify a patient’s hematopoietic cells via genome editing and is approved for the treatment of SCD in patients 12 years of age and older with recurrent vaso-occlusive crises. CRISPR/Cas9 can be directed to cut DNA in targeted areas, followed by editing it where it was cut. The modified stem cells are transplanted back into the patient, where they are engrafted within the bone marrow, increasing the production of fetal hemoglobin and preventing the sickling of red blood cells.

Lyfgenia uses a lentiviral vector for genetic modification wherein the patient’s blood stem cells are genetically modified to produce HbA^T87Q^, gene-therapy-derived hemoglobin that functions similarly to HbA, and these modified stem cells are then delivered to the patient.

Both these therapies are obtained from the patient’s stem cells, which are modified and infused back as a single dose infusion as a part of a hematopoietic stem cell transplant.

## 11. Implications for Healthcare

In general, the healthcare cost calculation for SCD is complex as the course of SCD is highly individualized, with multiple events occurring in a lifetime. The disease not only incurs medical costs but also leads to significant income and productivity loss [101].

A study by Lubeck et al. [16] found that individuals with SCD have a life expectancy of 54 years compared to 76 years for those without SCD. The quality-adjusted life expectancy was also lower for individuals with SCD, at 33 years old, compared to 67 years old for the non-SCD cohort. The study further calculated that due to the 22-year difference in life expectancy, there was a loss of approximately $695,000 in lifetime income for those with SCD. These findings are supported by Ballas et al. [101], who calculated that the expenditure for the care of SCD patients aged 19–50 was USD 7,393,600, with a life expectancy of 50 years.

In a study by Yeruva et al. [28], they inferred that patients with SCD who had CKD had a 1.6-day increase in hospital stay, and those with acute kidney injury and SCD had to lengthen their stay by 3.2 days. Correspondingly, the expenditure increased by USD 3866 in the CKD group and USD 8205 in the AKI group.

Johnson et al. [102] examined the lifetime burden of SCD on health systems and individuals and found that surviving individuals incurred an average of USD 34,477/year in SCD-attributable medical costs. The payers bore 96% of this cost. The total cost for the whole lifetime was USD 1.6 million per female patient and USD 1.7 million for male patients. The corresponding out-of-pocket total lifetime estimates were USD 42,395 per female patient and USD 45091 per male patient.

In a recent study, Aaron et al. [103] suggested a healthcare model to calculate the cost-effectiveness of the newer therapies in SCD. They calculated the probability of each possible outcome (including both acute and chronic conditions) based on insurance claims (Medicare or Medicaid) in the context of a specific socio-demographic group. Depending on the number of events that a patient experiences, the cost for that cycle and QALYs were integrated into a cost model. This model can be applied to justify the newer therapies and will provide payers and policymakers with an estimate of the average spending.

## 12. Conclusions

SCD remains a challenging medical condition with several systemic complications, including painful crises, pulmonary hypertension, thrombosis, and CKD, to name a few. SCD imposes a large economic burden on healthcare. A deeper understanding of the mechanism of end-organ damage and mitigation is warranted and remains a major unmet need in SCD. Given the socio-economic factors entangled with the care of SCD, prioritizing sustained healthcare delivery and equitable access to care for all individuals affected by this complex disease will help reduce the disease burden on society and healthcare costs and minimize the productivity loss due to illness. Increased awareness, education, and healthcare services can also enhance early detection, ultimately improving patient outcomes and their quality of life. Although the current therapeutic landscape is predominated by supportive care and the use of agents such as hydroxyurea, the latest therapeutic advances provide hope for a potential cure for SCD.

## Figures and Tables

**Table 1:** Racial demographics of the whole group (reference [7]).

Demographic Race	%
Black or African American	93.5
Caucasian	3.4
Asian	0.3
Latino or Hispanic	2.4
Native American	0.3
Middle Eastern	0.2

**Table 2:** Racial background of each country (reference [7]).

Race	Ghana	US	UK	Italy
N = 877	%	%	%	%
Black	100	90.9	91.5	76.5
Asian	0	0.4	0	2.5
Caucasian	0	3.1	2.9	21
Arab	0	0.8	0	0
Hispanic	0	4.3	5.6	0
Other	0	1.2	0	0

**Table 3:** Racial and ethnic differences prevalent amongst hospitalized patients with SCD in the US (reference [8]).

Ethnicity	Prevalence
Blacks	69,889 (93.4%)
Hispanics	3603 (4.8%)
Whites	1325 (1.8%)
Total	74,817
